# Tongluo Zhitong Prescription Alleviates Allodynia, Hyperalgesia, and Dyskinesia in the Chronic Constriction Injury Model of Rats

**DOI:** 10.1155/2017/8197281

**Published:** 2017-12-05

**Authors:** Zhiyong Wang, Jianwei Wang, Lihua Qin, Weiguang Zhang

**Affiliations:** ^1^Department of Anatomy and Histo-Embryology, School of Basic Medical Sciences, Peking University, Beijing 100191, China; ^2^Key Lab for Neuroscience, Ministry of Education/National Health and Family Planning Commission of China, Peking University, Beijing 100191, China

## Abstract

Neuropathic pain is common in clinical practice. Exploration of new drug therapeutics has always been carried out for more satisfactory effects and fewer side-effects. In the present study, we aimed to investigate effects of Tongluo Zhitong Prescription (TZP), a compounded Chinese medicine description, on neuropathic pain model of rats with chronic constriction injury (CCI). The CCI model was established by loosely ligating sciatic nerve with catgut suture, proximal to its trifurcation. The static and dynamic allodynia, heat hyperalgesia, mechanical allodynia, cold allodynia, and gait were assessed. Our results showed that TZP alleviated CCI-induced static and dynamic allodynia, suppressed heat hyperalgesia and cold and mechanical allodynia, and improved gait function. These results suggest that TZP could alleviate neuropathic pain. Further experiments are needed to explore its mechanisms.

## 1. Introduction

Neuropathic pain is caused by disease or injury of the nervous system, it affects up to 8% population [1]. Allodynia and hyperalgesia are prominent symptoms in patients with neuropathic pain, experienced either simultaneously or independently [2]. Current treatments include nonpharmacological, pharmacological, and interventional therapies for pain relief. Pharmacotherapy for neuropathic pain is the main treatment, and currently recommended first-line treatments include antidepressants and anticonvulsants [1]. However, there are many unsatisfactory aspects about these drugs, such as the limited pain relief and the occurrence of side-effects [3–5].

Traditional Chinese medicine is widely used to treat various diseases because of the fewer side-effects and may be effective in targeting different pathological mechanisms involved in peripheral nerve injury, avoiding the shortcomings of single factor adjuvant therapy [6]. TZP consists of* Astragalus*,* Salvia*, Scorpion, and* Asarum*.* Astragalus* and* Salvia* could promote the recovery of nerve structure and motor function [7, 8]; Scorpion and* Asarum* help to restore the sensory function [9, 10]. The prescription containing these four herbs mentioned above is expected to alleviate allodynia, hyperalgesia, and dyskinesia of the CCI model in present experiment.

In this study, we evaluated TZP using a neuropathic pain model of rats with CCI [11]. With several behavioral testing paradigms for allodynia, hyperalgesia, and dyskinesia, we found that TZP could alleviate neuropathic pain in rats.

## 2. Materials and Methods

### 2.1. Drugs

The composition of TZP was as follows:* Astragalus* 100 g,* Salvia* 50 g, Scorpion 20 g, and* Asarum* 10 g.

All medicines were purchased from Beijing, China. They were prepared according to the previous protocol [12], firstly being mixed and then boiled with 10 volumes of distilled water for 1 h. The boiling was carried out twice, and the mixed solution was concentrated to 1 g/ml and stored at 4°C until use.

### 2.2. Animals

Adult male SD rats weighing about 250 grams were raised under pathogen-free laboratory conditions on a 12 h light/dark cycle at about 23°C. Rats were divided into three groups: sham group, control group, and treatment group (6 rats in each group). The animals were allowed free access to food and water. Efforts were made to minimize possible unnecessary animal sufferings; experimental procedures on animals were performed according to the Guidelines for the Care and Use of Laboratory Animals of Peking University. The study protocol was approved by the Medical Ethics Review Committee of Peking University (Beijing, China).

### 2.3. CCI Model

CCI model was made according to described methods [11]. Rats in the control and treatment groups were anesthetized with Ketamine (80 mg/kg) and Xylazine (10 mg/kg). The right sciatic nerve was exposed at the level of the trifurcation. Four ligatures (chromic catgut suture 4/0) were placed loosely around the nerve, 1 mm apart, and proximal to the trifurcation of the sciatic nerve. Ligatures were tied; the constriction to the diameter of the sciatic nerve was barely discernable. When a brief twitch was observed, the constriction was immediately stopped. In the sham group, the nerve was exposed without ligation. Finally, the incisions were sutured.

### 2.4. Method of Administration

Starting from the first day after surgery, rats in the treatment group were treated with 2 ml TZP solution (1 g/ml) by oral gavage once daily, while rats in the sham and control groups were treated with 2 ml 0.9% NaCl in the same manner.

### 2.5. Static Mechanical Allodynia

Before behavioral assessment, the rat was allowed to acclimate for 1 h daily in the test environment for 5 days. The rat was placed on an elevated wire mesh bottom table with Perspex cage and habituated to the environment for 15 min before the experiment. Von Frey filaments (Stoelting, USA) were applied to the central plantar surface of the right hind paw. The 50% PWT in response to a series of von Frey filaments (0.41–15.1 g) was measured by the “up and down” method as described by Chaplan et al. [13].

### 2.6. Dynamic Mechanical Allodynia

The rat was habituated as above in the “static mechanical allodynia.” A cotton bud was used to lightly stroke the central plantar surface of the right hind paw. It was considered as a withdrawal response when the rat lifted or licked the paw; then the time taken to show a withdrawal reaction was recorded as the paw withdrawal latency (PWL). A cut-off time of 15 s was imposed [14].

### 2.7. Heat Hyperalgesia

The rat was allowed to acclimate for a minimum of 45 min within acrylic enclosures on a clear glass plate maintained at 40°C. A radiant heat source was focused onto the central plantar surface of the right hind paw. Measurements of PWL were taken by a timer that was started by the activation of the heat source and stopped when withdrawal of the paw was detected with a photodetector. The paw withdrawal latency (PWL) was recorded with a minimal value of 0.5 s and a maximum of 30 s. Three measurements were taken for each right hind paw and were averaged as a result of each test session [15].

### 2.8. Mechanical Hyperalgesia

Rats were placed on a cage with grid floor; a safety pin was used to press the central plantar surface of the right hind paw, so that the surface of the paw was briefly stimulated at an intensity sufficient to dimple but not penetrate it. Paw withdrawal duration (PWD) was recorded, with an arbitrary minimal time of 0.5 s and a maximal cut-off of 15 s [16].

### 2.9. Cold Allodynia

A drop of acetone solution was delicately dropped onto the central plantar surface of the paw, using a blunt needle connected to a syringe without touching the skin. The duration of the withdrawal response was recorded with an arbitrary minimal value of 0.5 s and a maximum of 15 s [17].

### 2.10. Motor Function

Sciatic functional index (SFI) was measured at 1 day before surgery and 1, 3, 5, 7, 14, 21, and 28 days after surgery as described previously [12]. The animals were made to walk in one direction through an acrylic corridor (70 cm × 15 cm) three times. The first three analyzable walks were evaluated. Black ink and white paper were used. The animals' hind feet were pressed onto an ink pad. After the rats walked through the corridor over the white paper, their footprints were measured with a ruler and rounded to the nearest 0.5 mm.

Print length (PL), toe spread (TS), and intermediary toe spread (IT) were measured. The left foot parameters were recorded as NPL, NTS, and NIT; the right foot parameters were recorded as EPL, ETS, and EIT. Sciatic function index (SFI) was calculated by the following formula: SFI = −38.3([EPL − NPL]/NPL) + 109.5([ETS − NTS]/NTS) + 13.3([EIT − NIT]/NIT) − 8.8.

In the above-described measuring methods, such as static mechanical allodynia, dynamic mechanical allodynia, heat hyperalgesia, mechanical hyperalgesia, cold allodynia, and motor function, there was an interval of two hours between any two tests, based on the purpose of avoiding interference with data accuracy.

### 2.11. Statistical Analysis

Results are presented as mean ± SE. The data were analyzed using two-way analysis of variance (ANOVA). A value of *P* < 0.05 was considered statistically significant.

## 3. Results

### 3.1. Static Mechanical Allodynia

**Figure 1:**
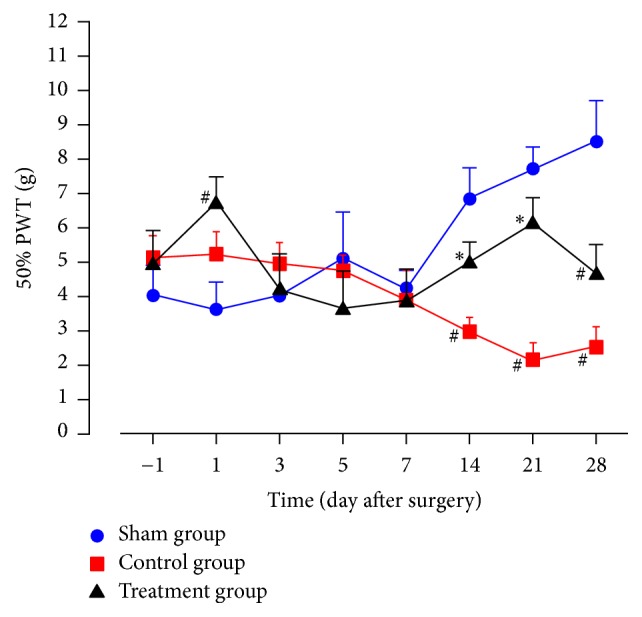
Static mechanical allodynia (^#^*P* < 0.05 compared to sham group; ^*∗*^*P* < 0.05 compared to control group).

As shown in Figure 1, from 14 to 28 days after surgery, 50% PWTs in the control group were significantly lower than in the sham group (*P* < 0.05), and 50% PWTs in the treatment group were significantly higher than those in the control group (*P* < 0.05), while the values in the sham and treatment groups were not statistically different. From 1 day before surgery to 7 days after surgery, the values between control group and treatment group were not statistically different.

### 3.2. Dynamic Mechanical Allodynia

**Figure 2:**
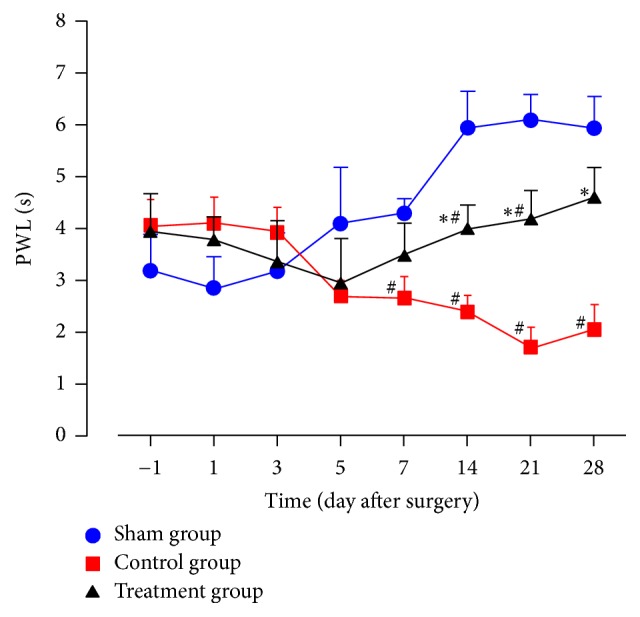
Dynamic mechanical allodynia (^#^*P* < 0.05 compared to sham group; ^*∗*^*P* < 0.05 compared to control group).

As shown in Figure 2, from 1 day before surgery to 5 days after surgery, PWLs between any two groups were not statistically different. From 7 to 28 days after surgery, PWLs in the control group were significantly lower than in the sham group (*P* < 0.05). From 14 to 28 days after surgery, the values in the treatment group were statistically higher than in the control group (*P* < 0.05). From 14 to 21 days after surgery, the values in the treatment group were statistically lower than in the sham group (*P* < 0.05).

### 3.3. Heat Hyperalgesia

**Figure 3:**
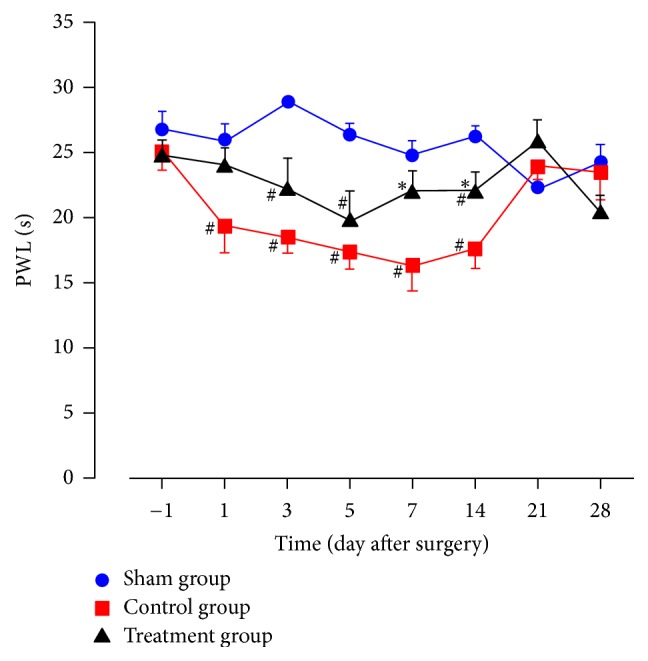
Heat hyperalgesia (^#^*P* < 0.05 compared to sham group; ^*∗*^*P* < 0.05 compared to control group).

Three, 5, and 14 days after surgery, PWLs in the treatment group were significantly lower than in the sham group (*P* < 0.05). From 1 to 14 days after surgery, PWLs in the control group were significantly lower than in the sham group (*P* < 0.05). 7 and 14 days after surgery, PWLs in the treatment group were significantly higher than in the control group (*P* < 0.05). 1 day before surgery and 21 and 28 days after surgery, PWLs between any two groups were not statistically different (Figure 3).

### 3.4. Mechanical Hyperalgesia

**Figure 4:**
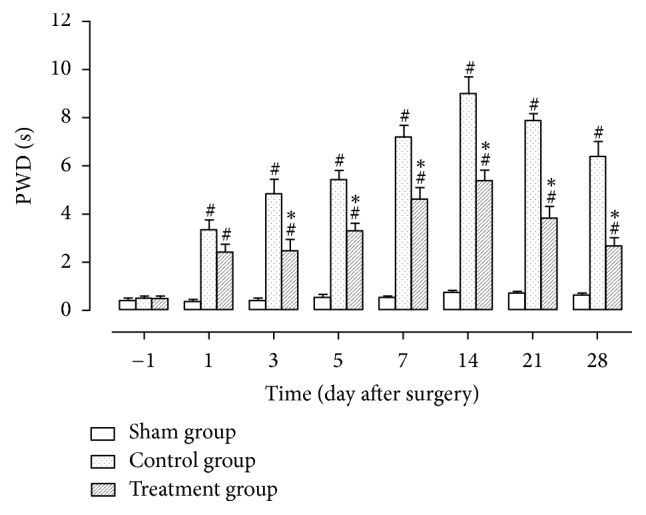
Mechanical hyperalgesia (^#^*P* < 0.05 compared to sham group; ^*∗*^*P* < 0.05 compared to control group).

One day before surgery, PWDs between any two groups were not statistically different. One day after surgery, PWDs in the control and treatment groups were significantly higher than in the sham group (*P* < 0.05), while the values between the two surgical groups were not statistically different. From 3 to 28 days after surgery, PWDs in the control and treatment groups were significantly higher than in the sham group (*P* < 0.05), while the values in the treatment group were significantly lower than in the control group (*P* < 0.05) (Figure 4).

### 3.5. Cold Allodynia

**Figure 5:**
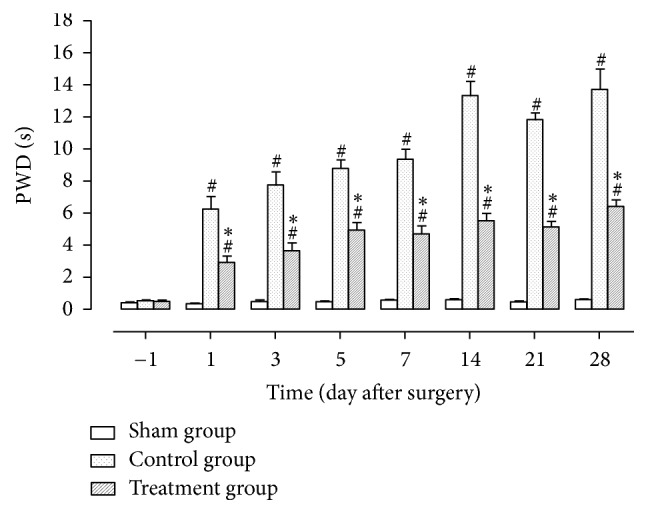
Cold allodynia (^#^*P* < 0.05 compared to sham group; ^*∗*^*P* < 0.05 compared to control group).

One day before surgery, PWDs between any two groups were not statistically different. From 1 to 28 days after surgery, PWDs in the control and treatment groups were significantly higher than in the sham group (*P* < 0.05), while the values in the treatment group were significantly lower than in the control group (*P* < 0.05) (Figure 5).

### 3.6. Motor Function

**Figure 6:**
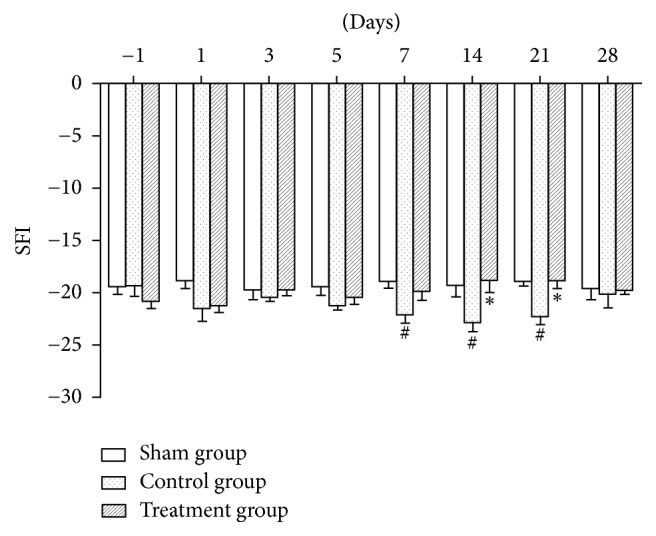
Motor function (^#^*P* < 0.05 compared to sham group; ^*∗*^*P* < 0.05 compared to control group).

From 1 day before surgery to 5 days after surgery, SFIs between any two groups were not statistically different. Seven days after surgery, SFIs in the control group were significantly lower than in the sham group (*P* < 0.05). Fourteen and 21 days after surgery, the values in the control group were significantly lower than in the sham group (*P* < 0.05), and the values in the treatment group were significantly higher than in the control group (*P* < 0.05), while the values between the sham and treatment groups were not statistically different. Twenty-eight days after surgery, the values between any two groups were not statistically different (Figure 6).

## 4. Discussion

The CCI model is a classical neuropathic pain model and resistant to many agents used for the symptomatic treatment of chronic neuropathic pain. In this study, we adopted the CCI model to study the role of TZP in allodynia, hyperalgesia, and dyskinesia.

The main treatment for peripheral neuropathic pain is pharmacotherapy; currently recommended drugs include antidepressants and anticonvulsants [1]. A series of compounds, such as morphine, cannabinoids D9-tetrahydrocannabinol, gabapentin, carbamazepine, and baclofen, has been used to modulate neuropathic allodynia and other manifestations of neuropathic pain [18]. But the occurrence of side-effects about these drugs is unsatisfactory, so it is particularly necessary to explore alternative medicines.

Traditional Chinese medicine has slight side-effects. In the present study, a Chinese prescription, TZP, was adopted to be applied to CCI rat model. By the theory of traditional Chinese medicine, in TZP,* Astragalus* and* Salvia* could promote regeneration of nerve fibers [7, 8]. Regenerated fibers are the structural basis for restoring normal sensory and motor function; thus the above two herbs are speculated to alleviate allodynia, hyperalgesia, and dyskinesia by promoting fiber regeneration in CCI model. Scorpion and* Asarum* help to restore the sensory function in other animal models [9, 10]; we speculated that the two herbs could improve allodynia and hyperalgesia in CCI model. This experiment was designed based on the above findings.

As shown in experimental results, TZP exerted analgesic effects on static mechanical allodynia, dynamic mechanical allodynia, heat hyperalgesia, mechanical hyperalgesia, cold allodynia, and dyskinesia. TZP alleviated CCI-induced neuropathic pain as evidenced by increased 50% PWTs (days 14–21, *P* < 0.05, Figure 1), increased PWLs reacting to light brushing (days 14–28, *P* < 0.05, Figure 2), increased PWLs reacting to heat stimuli (days 7–14, *P* < 0.05, Figure 3), reduced PWDs reacting to pin prick (days 3–28, *P* < 0.05, Figure 4), and reduced PWDs reacting to acetone (days 1–28, *P* < 0.05, Figure 5) compared to the control group. In terms of motor function, TZP improved SFI (days 14–21, *P* < 0.05, Figure 6) compared to the control group. The above results of this study demonstrated that TZP exerted analgesic effects to a certain extent in recovery of both sensory and motor functions in CCI model.

The mechanisms by which TZP treats the CCI model need further investigation. Here we just give some clues. According to previous studies, astragaloside IV might upregulate growth-associated protein 43 (Gap-43) expression in order to promote sciatic nerve regeneration and functional recovery [7]. Astragaloside IV might exert anti-inflammatory effects by inhibiting toll-like receptor 4 (TLR4) signaling pathway and nucleotide binding oligomerization domain-like receptors P3 (NLRP3) inflammasome overactivation in order to prevent transient cerebral ischemia and reperfusion injury [19]. In present study,* Astragalus* might protect the corresponding neurons in the spinal cord by the same mechanism, which need to be further verified. SalA might improve glucose metabolism through regulation of the AMPK-PGC1*α*-Sirt3 axis, in order to improve peripheral nerve function [8]. Salvianolic acid B promotes the recovery of neurological function and might be attributed to inhibiting apoptosis and promoting the differentiation and maturation of oligodendrocyte precursor cells [20].* Salvia* may promote functional recovery through the molecular mechanisms mentioned above. Indian red scorpion venom increases repolarization time and refractory period of the action potential greatly [21], while yellow Iranian scorpion,* Odontobuthus doriae* (*O. doriae*), could depress resting membrane potential 3 (RMP3) [22].* Scorpio* alleviating allodynia and hyperalgesia is likely to be associated with changes in membrane potential in this experiment, which need to be further researched. In a previous study,* Asarum* causes acute respiratory disturbance, associating with *β*-asarone-induced inhibition of neurotransmission in the medullary respiratory neuronal network [23].* Asarum* might improve paresthesia through changes of neurotransmission in the present experiment.

In this study, the analgesic effects of TZP were initially explored on CCI-induced neuropathic pain models, demonstrating that TZP could alleviate allodynia, hyperalgesia, and dyskinesia to a certain extent. Therefore, in the future, TZP might be an alternative option for the clinical management of some neuropathic pain, following elucidating its mechanism of action and the active component.

In addition to CCI model, there are other neuropathic pain models, such as spinal nerve ligation model (SNL) model, partial sciatic nerve ligation (PSL) model, and spared nerve injury (SNI) model, which need to apply TZP to further verify its efficacy.

In summary, using CCI model of neuropathic pain, we evaluated the analgesic effects of TZP, a Chinese medicinal description. This preliminary observation gives us a clue for the alternative option in the treatment of neuropathic pain.
